# NK Cell Therapy: A Rising Star in Cancer Treatment

**DOI:** 10.3390/cancers13164129

**Published:** 2021-08-17

**Authors:** Nawen Du, Feifei Guo, Yufeng Wang, Jiuwei Cui

**Affiliations:** Cancer Center, The First Hospital of Jilin University, 71 Xinmin Street, Changchun 130021, China; dunw18@mails.jlu.edu.cn (N.D.); guoff19@mails.jlu.edu.cn (F.G.); Yufeng_Wang@jlu.edu.cn (Y.W.)

**Keywords:** natural killer cell, immunotherapy, cell therapy, tumor microenvironment

## Abstract

**Simple Summary:**

A cancer treatment approach known as immunotherapy has become popular in the medical field. In this case, immune cells are boosted for effective response against cancer. A type of immune cell with significant potential for use in immunotherapy is the natural killer (NK) cell. The number of NK cells in the cancer tissues has been shown to be lower than normal, and this contributes to the growth of cancer cells. Besides, the immune function of the NK cells is compromised, thus interfering with anticancer immunity. Many research studies are being conducted to develop cancer treatment strategies based on increasing the number of NK cells and enhancing their activity.

**Abstract:**

Immunotherapy has become a robust and routine treatment strategy for patients with cancer; however, there are efficacy and safety issues that should be resolved. Natural killer (NK) cells are important innate immune cells that have attracted increasing attention owing to their major histocompatibility complex-independent immunosurveillance ability. These cells provide the first-line defense against carcinogenesis and are closely related to cancer development. However, NK cells are functionally suppressed owing to multiple immunosuppressive factors in the tumor microenvironment; thus, releasing the suppressed state of NK cells is an emergent project and a promising solution for immunotherapy. As a result, many clinical trials of NK cell therapy alone or in combination with other agents are currently underway. This review describes the current status of NK cell therapy for cancer treatment based on the effector function and releasing the inhibited state of NK cells in the cancer microenvironment.

## 1. Introduction

In the past decade, cancer immunotherapy has undergone a renaissance, especially the successes of immune checkpoint blockade and chimeric antigen receptor (CAR)-T cell therapy. Although durable clinical remissions have been observed, only a few patients have benefited, and adverse effects are still a concern. Natural killer (NK) cells are key components of the innate immune system. Several clinical studies have confirmed the close relationship between NK cells and cancer development. Individuals with lower NK cell cytotoxicity are more susceptible to cancer [1]. In addition, individuals with higher expression of activating receptors and higher NK cell cytotoxicity have a reduced risk of cancer and a more favorable prognosis [2,3]. These facts underscore the essential role of NK cells in cancer immunosurveillance.

Compared with other immunotherapies, NK cell therapy has its advantages. With their major histocompatibility complex (MHC)-antigen stimulating independent cytotoxicity, better safety, and high feasibility for “off-the-shelf” manufacturing, NK cells show great potential for treating cancers. However, the number and activity of NK cells are generally suppressed in cancer patients, and the function of NK cells is affected by a series of inhibitory factors in the cancer microenvironment; thus, strategies focusing on recovering the effector function of NK cells are under investigation in clinical trials. In addition to NK cell infusion as a single therapy, various optimized methods according to each aspect of the process, including source, expansion, persistence, targeting, and cytotoxicity of the NK cells, have been explored and demonstrated. Moreover, NK cell infusion combined with other compounds, including cytokines and antibodies, shows better and safer therapeutic effects. Therefore, this review discusses the mechanism of NK cells in cancer treatment and the current situation of NK cell therapy in clinical practice.

## 2. An Overview of NK Cell Biology and Functions

Human NK cells originate from CD34+ hematopoietic stem cells in the bone marrow and widely spread throughout lymphoid and non-lymphoid tissues, such as blood, liver, uterus, and spleen [4]. Analysis of NK cell properties and phenotypes has demonstrated functional heterogeneity in NK cell populations with different maturation stages and anatomical locations. NK cells at different maturation stages and functional features are distinguished through the expression of receptors [5]. CD56, an important marker expressed on NK cells, provides a functional classification of NK cells.

Human NK cells can be divided into CD56bright and CD56dim subsets, and both subsets participate in anti-cancer immunity with distinct functional role as described below. Most immature NK cells primarily transition into a minor CD56bright population (about 5%), then downregulate CD56 expression and converts into the major CD56dim population (>90%), which begins to express CD16 (FcγRIII) and acquires potent cytotoxicity [6]. Naive CD56bright NK cells reside primarily in tonsils, spleen, and lymph nodes, and tissue-resident NK cells are predominantly CD56bright.

As mediators of the innate immune system, NK cells have two major functions: cytotoxic effect and immune regulation. Without pre-activation, NK cells can recognize and kill abnormal cells by releasing perforin and granzymes [7]. In addition, stimulation of NK cells by killer activating receptors (KARs) induces the expression and release of death ligands, including tumor necrosis factor-alpha (TNFα), Fas ligand (FasL), and TNF-related apoptosis-inducing ligand (TRAIL), triggering the apoptotic pathway [8]. As regulatory cells, NK cells secrete a set of cytokines and chemokines, including interferon-gamma (IFN-γ), interleukin (IL)-10, CCL3, CCL4, CCL5, and lymphotactin, that bridge the innate and adaptive immunity [9].

NK cell activity is precisely regulated by receptors expressed on NK cells. Positive signal activating receptors and negative signal inhibitory receptors jointly mediate the outcome of NK cell-target cell encounters (Table 1) [10]. Many NK cell inhibitory receptors are specific for MHC class I molecules, which are expressed nearly by every healthy cell but are absent on infected or cancer cells. Through the recognition of self-molecules, MHC-I, NK cells detect “missing self” using their inhibitory receptors, killer immunoglobulin-like receptors (KIRs)(human)/Ly49(mouse) [11]. Another inhibitory receptor, the CD94-NKG2A heterodimer, is expressed in both human and mice species. With the immunoreceptor tyrosine-based inhibition motif in the cytoplasmic tail, NKG2A triggers an inhibitory signal after binding to the non-classical human leukocyte antigen (HLA) class I molecule, HLA-E, which is normally expressed by all cells [12]. In addition, inhibitory leukocyte immunoglobulin-like receptor-1 (LIR-1) is also expressed on NK cells binding to HLA-G, which complements KIR and CD94/NKG2A in the recognition of HLA class I molecules [13]. Other inhibitory receptors including TIGIT, CD96, LAG3, and PD-1, are critical for maintaining NK cell self-tolerance. However, these receptors are upregulated as cancer progression leading to the exhausted status of NK cells. These “checkpoints” are druggable targets in cancer immunotherapy [14].

NKG2D and DNAM-1 (CD226) are two common activating receptors expressed on NK cells. The ligands of NKG2D include MHC class I homologues MICA/B and ULBP (cytomegalovirus UL16-binding protein) in humans and RAE1 (retinoic acid early transcript 1), MULT1 (murine UL16-binding protein-1), and H60 in mice [15]. After recognizing these ligands, NKG2D receptors execute function via the adaptor protein DAP10, which recruits phosphatidylinositol-3 kinase and triggers cytotoxicity [16]. DNAM-1 binds to CD112 (also known as nectin 2) and CD155 (also known as the poliovirus receptor, PVR), which is improperly expressed in cancer cells [17]. CD96 and TIGIT expressed on NK cells can competitively bind to these two ligands and negatively modulate NK cell activity, including limiting cytotoxicity and restricting IFN-γ production. Notably, the expression of DNAM-1 ligands by cancer cells is associated with low levels of NKG2D ligands, which means NK cell-mediated killing is triggered by either the DNAM-1 or NKG2D pathway. Such immune selection is related to the surface phenotype of cancer cells [18].

Another potent activating receptor is CD16. Following crosslinking with the Fc region of antibodies, CD16 triggers antibody-dependent cellular cytotoxicity (ADCC) against antibody-coated cancer cells [19]. ADCC is triggered by several therapeutic antibodies recognizing tumor-associated antigens with increased NK cell cancer infiltration, suggesting a cancer-homing effect, and this demonstrates the efficacy of NK cells in adaptive immunity [20]. Immunoglobulin superfamily receptors, NKp46, NKp30, and NKp44, are collectively called natural cytotoxicity receptors and are implicated in the recognition and lysis of viral, bacterial, and cancer cells [21,22,23,24]. Among these receptors, only NKp44 is selectively expressed by IL-2 activated NK cells while NKp46 and NKp30 are expressed by all NK cells [25]. These receptors trigger NK cells’ cytokine production and cytotoxicity directly.

## 3. NK Cells Are Essential for Defense against Carcinogenesis

**Figure 1:**
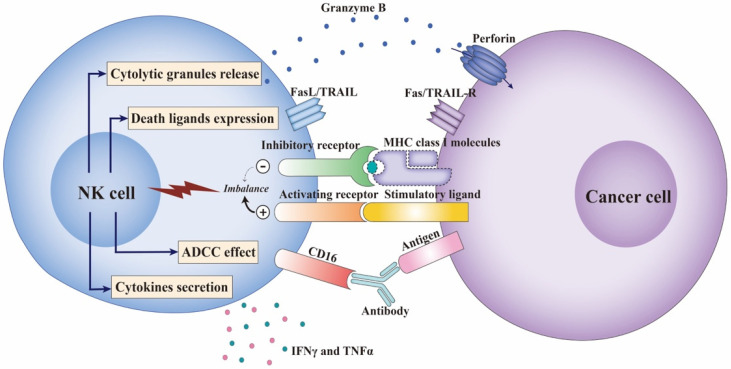
The function of NK cells in response to cancer cells. The activity of NK cells is regulated by the signal input from the activating and inhibitory receptors expressed on NK cells. Cancer cells upregulate stimulatory ligands for NK cell-activating receptors such as NKG2D and downregulate MHC class I molecules to avoid cytotoxic T cell killing, which breaks the balance and leads to NK cell activation. Activated NK cells rapidly synthesize and release cytolytic granules, perforin and granzymes, initiating cancer cell apoptosis. NK cells can also express death ligands, FasL and TRAIL, combined with Fas and TRAIL-R expressed on cancer cells, to mediate apoptosis. A specific function of NK cells in anti-cancer immunity is to exert ADCC by expressing CD16 to recognize antibody-coated cancer cells. In addition, as immunoregulatory cells, NK cells secrete a set of cytokines and chemokines, especially IFN-γ and TNFα, which could promote NK cell anti-cancer function and stimulate other maturation and activation of other lymphocytes. ADCC-Antibody-dependent cellular cytotoxicity; IFN-γ-Interferon-gamma; MHC-Major histocompatibility complex; NK cell-Natural killer cells; TNFα-Tumor necrosis factor-alpha; TRAIL-R-TNF-related apoptosis-inducing ligand.

In patients with leukemia undergoing allogeneic stem cell transplantation, NK cells are the first lymphocytes to appear and react before T cell exercises its function [26]. In addition, individuals with higher levels of NK cell cytotoxicity have a low incidence of cancer, proving the potent immunosurveillance ability of NK cells [27]. CD56dim subsets constitute the majority of NK cells in circulation (blood, lung, and spleen) with stronger cytotoxicity and higher responsiveness to stimulation by cancer cells. Despite their relatively low abundance, CD56bright NK cells have a strong capacity for cytokine and chemokine production in response to stimulation by IL-12, IL-15, and IL-18. Moreover, through producing IFN-γ and chemokines, NK cells promote the maturation and activation of dendritic cells, macrophages, and T cells performing their anticancer functions [9]. Such immunoregulatory properties demonstrate the essential role of NK cells in the early immune response to carcinogenesis (Figure 1).

Circulating NK cells are recruited to the sites of carcinogenesis by chemokines secreted by lymphocytes, and the effector function is triggered by the imbalance of transmitted signals from surface receptors [28,29]. Ligands for NK cell-activating receptors are usually poorly expressed on normal cells. However, in cancer cells, abnormal cell proliferation contributes to DNA replication stress and genomic instability, which finally induces the expression of NKG2D and DNAM-1 ligands in stressed cells [30]. The expression of activating receptors NKG2D and DNAM-1 on NK cells also modulate their lysis capability in patients with acute myelogenous leukemia (AML), myelodysplastic syndrome, multiple myeloma, and ovarian carcinoma [18,31,32,33]. In addition, ADCC is triggered in response to CD16 recognition. Different allotypes of IgG-Fc-receptor trigger differential activity of NK cell-mediated ADCC, which could predict the outcome in clinical trials [34]. Strategies enhancing NK cell-mediated ADCC, including Fc fragment modification or antibody engineering, are under investigation as therapeutic options [35,36]. Prevention of CD16 shedding or combination with agents targeting co-receptors in NK cells is also being explored [37].

In addition, cancer cells lose or downregulate the expression of MHC I molecules to evade recognition by CD8+ T cells, which would break the inhibited condition of NK cells and consequently trigger their effector function. NK cells rapidly synthesize and release perforin and granzymes into the synaptic cleft, initiating apoptosis, whereas death ligand-mediated apoptosis occurs later [38]. In several trials, allogeneic NK cells mediate the lysis of hematological cancer cells, which is associated with better outcomes based on KIR-ligand mismatching. In addition, dual blockade of NKG2A and LIR-1 improves the cytotoxicity of KIR-NK cells and leads to significant killing of AML or acute lymphocytic leukemia cells [39]; thus, removing the inhibition of NK cells is required for cancer cell lysis.

NK cells are essential in resisting carcinogenesis, although they have poor infiltration and lower cytotoxic ability in the tumor microenvironment (TME). As the development and progression of cancer are correlated with the dysfunction of NK cells, enhancing the function of NK cells is necessary for anti-cancer immunity.

## 4. Immunosuppressive State of NK Cells in TME

In the TME, immunosuppressive cells and cytokines inhibit NK cell function. Moreover, dysfunctional NK cells can, in turn, adversely influence other anti-cancer processes, forming a vicious cycle. Understanding the mechanisms of the suppressed state of NK cells is useful for clarifying therapeutic targets.

### 4.1. Abnormal Ligand Expression of Cancer Cells Promote Immune Escape

When NK cells interact with cancer cells, the ligands on cancer cells for activating receptors trigger NK cell recognition and elimination. However, cancer cells shed NKG2D ligands through “a disintegrin and metalloproteinases” (ADAM) family, which could proteolytically cleave MICA resulting in a reduction of MICA surface density [40]. Therapeutic blockade of related ADAM proteases seems promising for preventing cancer progression. In addition, soluble ligands released by cancer cells, such as MICA/B, bind NKG2D, which blocks the interaction between NK cells and target cells, thus downregulating NKG2D expression on the surface of NK cells and promoting immune evasion [41]. Another shed NKG2D ligand, MULT1, was thought to be inhibitory; however, it was found that soluble MULT1 promotes NK cell activation and cancer rejection in vivo due to its high affinity for the NKG2D receptor, which reverses global desensitization [42].

### 4.2. Immunosuppressive Cytokines in TME

TGFβ is commonly viewed as the most potent immunosuppressive cytokine in the TME. After binding to NK cells, active TGFβ triggers the phosphorylation of Smad2/3, which represses the expression of T-bet, a positive stimulator of IFN-γ genes, limiting the secretion of IFN-γ [43]. Tumor cells-derived TGFβ could affect the chemokine receptor repertoire of NK cells [44]. In addition, Treg cell membrane-bound TGFβ and soluble TGFβ in TME inhibit NK cell function and downregulate the expression of NKG2D in NK cells [45]. Moreover, TGFβ inhibits the anti-cancer function of NK cells by repressing the mTOR pathway, which suppresses proliferation and granzyme production. The mTOR pathway is activated after exposure to high concentrations of IL-15 and positively affects the expression of IL-15 receptor, thus controlling the metabolic activity of NK cells [46]. Additionally, TGFβ promotes the expression of fructose-1,6-bisphosphatase (FBP1), which inhibits NK cell glycolytic metabolism, thus suppressing the activity of NK cells and mediating immune evasion [47]. TGFβ is considered a major target of inhibitory immune factors in cancer, and inhibitors of TGFβ/TGFβR are currently under clinical research. Another immunosuppressive cytokine IL-6 impairs the function of NK cells by activating the STAT3 pathway on NK cells, leading to the downregulation of activating receptors [48]. IL-10, despite being categorized as an immunosuppressive cytokine, enhances the production of IFN-γ in NK cells instead of inhibiting their function [49].

### 4.3. Immunosuppressive Metabolic Factors in TME

In terms of metabolism, cancer cells compete with activated NK cells for glucose and glutamine to produce ATP and sustain rapid cell growth. The Warburg effect causes the cancer environment to be acidic and oxygen-deficient, which diminishes IFN-γ secretion and inhibits the cancer surveillance function of NK cells [50]. Various treatment approaches targeting hypoxia and hypoxia-inducible factors, including hypoxia-activated prodrugs, improve the treatment efficacy of patients who are resistant to therapy [51]. A recent study based on single-cell RNA sequencing of tumor-infiltrating NK cells showed that inhibition of hypoxia-inducible factor-1α expression in NK cells could unleash their activity in solid tumors [52]. In addition, other metabolites such as indoleamine 2,3-dioxygenase, adenosine, and PGE2 can also suppress the maturation, proliferation, and functional activities of NK cells [53].

### 4.4. Interference by Other Immune Cells in TME

Immunosuppressive lymphocytes, including regulatory T cells (Treg cells), tumor-associated macrophages, myeloid-derived suppressor cells, cancer-associated fibroblasts, and tolerogenic dendritic cells, suppress the activity of NK cells by secreting immunosuppressive products (TGFβ and metabolites) or disrupt the interaction between NK cells and cancer cells through competition or misleading decoy [54]. Additionally, Treg cells secrete vesicles containing IL-37, a member of the IL-1 family, which suppresses the function of NK cells by changing the phenotype and depressing the function of NK cells [55]. In addition, secreted IL-37 binds to IL-18Rβ on NK cells, blocking the formation of the functional IL-18 receptor complex and thus inhibiting IL-18-induced IFN-γ production by NK cells [56]. Additionally, IL-37 binds to the negative checkpoint IL-1R8 (SIGIRR) expressed on NK cells. Inactivation of IL-1R8 unleashes the function of NK cells against carcinogenesis and metastasis [57]. Moreover, platelets coat circulating cancer cells, which suppresses NK cell activation by releasing TGFβ or displaying ligands for inhibitory receptors and disrupting activating ligand expression [58,59].

### 4.5. Dysfunctional NKreg Cells

In solid tumors, tumor-infiltrating CD56bright NK cells, which are also known as regulatory NK cells (NKreg), alter the phenotype of the immunosuppressive microenvironment. NKreg cells have a high expression of inhibitory receptor CD94-NKG2A and negatively express CD16, thus exhibiting lower cytotoxicity [60]. In addition, NKreg cells have immunomodulatory abilities, such as secreting IL-10 and TGFβ, and NKG2D and NKp46 receptors expressed on NKreg cells interact with the corresponding ligands expressed on T cells to suppress T cell proliferation and effector functions [61]. Therefore, comprehensive elucidation of the mechanism underlying the suppression of the anti-cancer immunity by the NKreg cells can inform the development of novel immunotherapy. CD73, a newly discovered checkpoint expressed on NKreg cells, is discussed below.

### 4.6. NK Cells Negatively Affect Anti-Cancer Immunity

Cancer-infiltrating NK cells sometimes show low cytotoxic potential and even angiogenic functions through the production of the angiogenic factor VEGFA. STAT5 is a key regulator that suppresses the secretion of VEGFA and restores the cytotoxicity of exhausted NK cells; thus, inhibitors targeting the JAK-STAT5 pathway could have pro-carcinogenesis potential [62]. In addition, in cancers derived from chronic inflammation, NK cells may possess pro-carcinogenic functions. NKG2D ligands in the cancer inflammatory environment recruit and activate CD8+ T cells. Activated CD8+ T cells and NK cells produce inflammatory cytokines, such as TNFα and IFN-γ, through the NKG2D/NKG2D-ligand pathway, leading to aggravated inflammation, which accelerates cancer progression [63]. Strategies targeting the NKG2D pathway may be useful for treating this type of cancer.

## 5. NK Cell Therapies

NK cells are essential for anti-cancer immunity. The presence of many inhibitory factors in the TME has been shown to suppress the function of NK cells. In addition, the biological characteristics of NK cells and modification techniques for improving the efficacy of NK cell therapies are widely discussed. Clinical trials using NK cell therapy alone or in combination with other approaches for treating cancer patients are ongoing and show promising therapeutic effects.

### 5.1. Allogeneic NK Cell Infusion Therapy

Adoptive transfer of autologous NK cells is considered a feasible approach, owing to the convenience of sourcing NK cells, lack of the requirement for immunosuppression, and the low risk of graft versus host disease. However, the increased number of circulating NK cells in the peripheral blood fails to produce the expected therapeutic response, perhaps due to the inhibition of self-HLA molecules [64]. In addition, compared with allogeneic NK cells, autologous NK cells are often obtained from heavily pretreated patients with limited expansion efficiency and cytotoxicity. Thus, these limitations restrict the continued exploration of autologous NK cell infusions.

In allogeneic NK cell infusion therapy, mismatches between inhibitory KIRs expressed on donor NK cells and recipients’ HLA ligands trigger alloreactivity of NK cells [65]. To avoid the immunologic rejection of allogeneic NK cells by the recipient, non-myeloablative chemotherapy is needed before adoptive transfer. Research has shown that AML patients with infusion of haploidentical NK cells following high-dose cyclophosphamide and fludarabine achieve complete remission. Notably, lymphodepleting conditioning treatment contributes to the remarkable increase in endogenous IL-15 levels essential for the expansion and persistence of donor NK cells [66].

**Figure 2:**
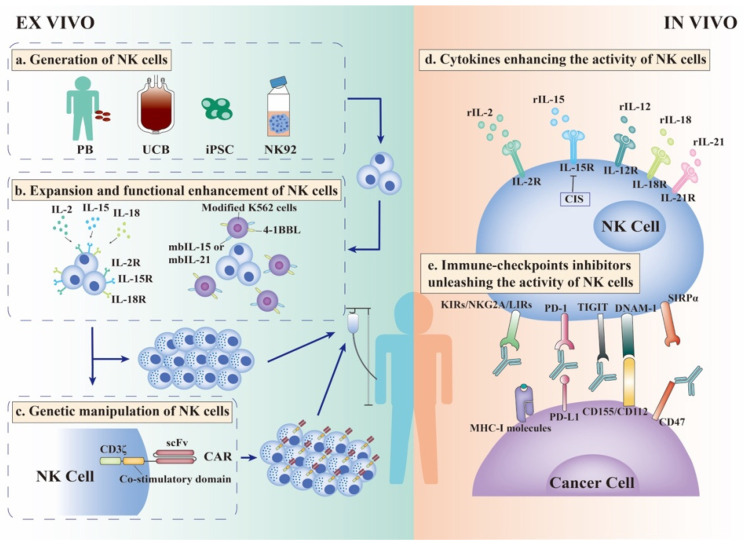
Strategies to enhance the efficacy of NK cell therapies. Allogeneic NK cell infusion is an efficient therapy for NK cells. For the best efficacy, a series of strategies are used to enhance the expansion and function of NK cells ex vivo. (**a**). NK cells can be widely generated from peripheral blood (PB), umbilical cord blood (UCB), induced pluripotent stem cells (iPSCs), and NK92 cell lines. (**b**). After isolation from these sources, NK cells must be stimulated by cytokines such as IL-2, IL-15, and IL-18 to empower NK cells with enhanced cytotoxicity and IFN-γ production ability. In addition, modified K562 cells, as feeder cells expressing membrane-bound IL-15, IL-21, and 4-1BBL, can induce greater proliferation and activation of NK cells. After stimulation by cytokines and/or feeder cells, haploidentical NK cells with sufficient quality and quantity can be infused into patients. (**c**). Moreover, NK cells can be modified ex vivo to express CARs, allowing NK cells to recognize specific tumor-associated antigens (TAA), which increase the targeting and efficacy of NK cell therapy. (**d**). In vivo, cytokines can stimulate the expansion and activation of NK cells, especially IL-2 and IL-15. (**e**). Immune-checkpoint inhibitors (ICIs) are used to relieve the inhibitory state of NK cells in vivo. Classical NK cell inhibitory receptors, KIRs, NKG2A, and LIRs, combine with MHC--I molecules expressed on cancer cells to exert “dominant inhibition” of NK cells. Antibodies against these receptors can unleash NK cells and enhance their anti-cancer ability. TIGIT is an inhibitory receptor expressed on NK cells that competes with the activating receptor DNAM-1 for ligands CD155/CD112 expressed on cancer cells. Therefore, blocking TIGIT could prevent the exhaustion of cancer-infiltrating NK cells and stimulate potent anti-cancer efficacy. Except for classical PD-1/PD-L1 signal, inhibitory ligands CD47 expressed on cancer cells interact with SIRPα expressed on NK cells and deliver “don’t eat me” signal into NK cells. The CD47 antibody combined with PD-L1 antibody could reverse the incapacity of NK cells and stimulate more effective anti-cancer immunity. CAR-Chimeric antigen receptor; iPSC-induced pluripotent stem cell; IL-Interleukin; NK Cell-Natural killer cells; PB-Peripheral blood; UCB-Umbilical cord blood.

Overall, various strategies to enhance the activity of NK cell infusion have been explored (Figure 2)**.**

#### 5.1.1. Ex Vivo Generation of NK Cells

NK cells can be generated from multiple sources, including peripheral blood, umbilical cord blood (UCB), NK cell lines, and induced pluripotent stem cells (iPSCs). By adding cytokines and feeder cells, the culturing environment of peripheral blood and UCB guarantees the purity, persistence, and expansion of NK cells ex vivo [67,68]. The NK92 cell line with high cytotoxicity and easily expanded characteristics can broadly kill cancer cells irrespective of cancer antigen; thus, it is considered a promising “off-the-shelf” source [69]. iPSC is considered for a standardized “off-the-shelf” therapy for any patient regardless of HLA haplotype. Undifferentiated iPSCs using cytokines and feeder cells (usually murine stromal cells) differentiated into CD34+ hemopoietic progenitor cells (HPCs), after flow sorting, these HPCs were transferred to the culture containing the cytokines IL-3, IL-15, IL-7, SCF, and Flt3L for 28-32 days to promote NK cell differentiation [70]. Based on this method, Hermanson et al. established a feeder-free, sorting-free approach to generate functional NK cells, which have been proven effective in treating mouse ovarian models [71].

#### 5.1.2. Ex Vivo Expansion and Functional Enhancement of NK Cells

Allogeneic therapy is required to generate NK cells of sufficient quality and quantity in vitro to perform infusion, and based on the biological characteristics of NK cells, several methods can be adopted. Cytokines are commonly used to stimulate NK cell activity. A recent clinical trial using IL-2-activated haploidentical NK cells as a bridge to hematopoietic stem-cell transplantation following cyclophosphamide/fludarabine chemotherapy in high-risk myelodysplastic syndrome patients showed complete remission, indicating the potential of NK cell therapy in treating refractory patients [72]. In addition, preactivation of NK cells with IL-12, IL-15, and IL-18 enhances IFN-γ production ability, which persists for weeks to months [73]. The enhanced proliferation of NK cells and IFN-γ production has been proven against ovarian tumor mouse models compared with those without cytokine pre-stimulation [74]. A phase I clinical study using this method to pretreat adoptively transferred NK cells into AML patients showed a clinical response in five of nine patients, among which four had complete remission [75].

Another strategy to stimulate the expansion of NK cells ex vivo is to utilize feeder cells, such as autologous peripheral blood mononuclear cells, Epstein-Barr virus-transformed lymphoblastoid cell lines, and genetically modified K562 cells. In particular, modified K562 cells expressing membrane-bound IL-15, IL-21, 4-1BBL, or OX40L induce greater proliferation and activation of NK cells [76]. These methods not only enhance NK cell expansion by thousand-fold but also induce higher expression levels of activating receptors, NKG2D and CD16, and superior cytokine secretion ability [77]. Moreover, the combination of cytokines and feeder cells can ensure more effective expansion and activation of NK cells [78].

#### 5.1.3. Ex Vivo Genetic Manipulation of NK Cells

CAR-T cell therapies have been approved for the treatment of acute lymphocytic leukemia and B-cell non-Hodgkin lymphoma. However, this therapy is limited by several shortcomings, including cytokine release syndrome, minimal isolation, transduction, and expansion, as well as inefficiency in solid tumors [79]. Given the innate biological properties of NK cells, CAR-NK cell therapies could circumvent these shortcomings. Compared with T cells, NK cells producing cytokines are less proinflammatory and safer. Liu et al. generated a CAR-NK cell derived from UCB transduced with a retroviral vector expressing genes encoding anti-CD19 CAR, IL-15, and an inducible caspase-9 suicide switch. CAR-NK treatment of CD19+ patients showed promising expansion, persistence, and outcomes without the development of any major adverse effects [80]. CAR-T cell therapies require the collection of autologous T cells and gene modification, which is both time-consuming and inefficient. In contrast, NK cells can be widely derived from several sources, as mentioned above. NK92 cells are easy to transduce and expand. However, NK92 derived CAR-NK cells require irradiation, which limits their survival and proliferation, while iPSC-NK cells can be produced from a standardized cell population that matches the clinical scale [81]. Using iPSCs for NK cell production provides more potential for gene modification, repeat dosing, and production of standardized products, which allows for more effective therapy against refractory solid tumors [82]. Li et al. [83] generated a novel iPSC-derived CAR-NK cell product targeting mesothelin containing the transmembrane domain of NKG2D, the 2B4 co-stimulatory domain, and the CD3ζ signaling domain to potentiate cancer cell killing. Mesothelin is a cell-surface antigen overexpressed in ovarian tumor and this CAR-NK product displayed superior anti-ovarian cancer activity and low toxicity in a mouse model [83]. Overall, many CAR-NK clinical trials are in progress to evaluate the efficacy and safety of this treatment approach.

### 5.2. Cytokines Enhancing the Activity of NK Cells In Vivo

Cytokines with immunomodulatory effects are essential to sustain the survival, proliferation, and maturation of NK cells. These cytokines include IL-2, IL-15, IL-12, IL-21, and IL-18; IL-2 and IL-15 are the most widely studied. They are required for NKG2D-mediated cytotoxicity and IFN-γ secretion; thus, they are broadly used as incubators before NK cell infusion or as infusing agents [84]. Although there are efficacy and safety concerns, various solutions are under investigation to optimize the treatment effect (Figure 2).

#### 5.2.1. Cytokine Treatment Based on IL-2

IL-2 is the first cytokine approved for use in patients owing to its promising ability to activate anti-cancer immunity and stimulate the production of lymphokine-activated killer cells [85]. However, the therapeutic effect is limited due to the high-affinity receptor IL-2Rα (CD25), which is competitively expressed on Treg cells, and severe side effects are observed due to activation of the vascular endothelial cells. Therefore, solving these shortcomings is required to improve IL-2 therapy. Researchers engineered a mutant form of IL-2 called “super-2” with increased binding affinity for IL-2Rβ, which avoided the interference of Treg cells and induced superior expansion as well as improved anti-cancer activities of NK cells [86]. Considering that IL-2Rβ-enhanced IL-2 mutants could not completely block the binding between Treg and IL-2, researchers designed an IL-2 fusion protein combining the high-affinity NKG2D ligand orthopoxvirus major histocompatibility complex class I like protein (OMCP) with IL-2 mutants, reducing the affinity for IL-2Rα. This fusion protein selectively activates NKG2D-bearing cells rather than IL2 Rα-bearing cells, which not only promotes the expansion and activation of NK cells but also avoids the adverse side effects caused by expanding Treg cells [87].

In addition to monotherapy, optimized IL-2 therapies combined with NK cell infusion therapy are under investigation for stimulating the activity of NK cells in vivo. In a clinical trial, IL-2-diphtheria toxin fusion protein (IL2DT) in adoptive NK cell infusion for the treatment of AML patients ablated Treg cells and showed improved clinical efficacy [88].

#### 5.2.2. Cytokine Treatment Based on IL-15

IL-15 is considered a crucial cytokine for sustaining NK cell proliferation, maturation, and functional activation. Based on anti-cancer and immunostimulation ability, recombinant IL-15 (rIL-15) was approved for testing in phase I trials treating refractory solid tumors through intravenous or subcutaneous administration. Without prior lymphodepletion, rIL-15 monotherapies could be safely administered and could induce a substantial increase in circulating NK and CD8+ T cells; however, no objective responses were observed. These results might be explained by the fact that patients in the clinical trials were heavily pretreated, and most amplified NK cells were relatively less cytotoxic [89]. Therefore, efficient approaches to enhance the bioactivity and persistence of IL-15 are required.

Heterodimeric IL-15 (hetIL-15) comprising IL-15 and IL-15Rα, which have an extended half-life and superior bioactivity, induce stronger persistence and expansion of NK and CD8+ T cells compared with rIL-15 [90]. In the absence of lymphodepletion, hetIL-15 administration enhances T cell and NK cell entry into tumors and improves the therapeutic outcome in solid tumor mouse models [91,92]. Another novel approach named N-803, an IL-15 super-agonist, comprises an IL-15 mutant (N72D) bound to the IL-15Rα-IgG1-Fc fusion protein. The mutation increases the binding affinity of IL-15 to IL-15Rβ and provides a nearly 30-fold increase in biological activity compared to IL-15. The Fc portion prolongs half-life and induces stronger immunological effects of the agonist in vivo [93]. N-803 therapies have been proven effective in reducing cancer progression and improving survival in several mouse models and are also being tested in multiple clinical trials [94]. In a phase Ib trial, patients with non-small-cell lung carcinoma (NSCLC) were treated with N-803 combined with immune checkpoint inhibitors, which showed safety and promising clinical activity [95]. Another trial showed that N-803 combined with anti-CD20 mAbs enhanced NK cell cytotoxicity in consideration of ADCC, resulting in a higher survival rate [96]. These trials illustrate the effectiveness of improving NK cell activity in different ways.

### 5.3. Immune-Checkpoint Inhibitors Unleashing the Activity of NK Cells In Vivo

Cancer cells suppress anti-cancer immunity by targeting immune checkpoints expressed on immune cells through cell-cell contact or exosome secretion. Typical immune-checkpoint PD-1/CTLA-4 inhibitors have been widely researched and applied in clinical practice because of their significant efficacy in relieving the inhibitory state of T cells. NK cells sustain functional homeostasis through activating and inhibitory receptors, and cancer cells upregulate ligands of inhibitory receptors to evade immunosurveillance. Therefore, blocking inhibitory checkpoints is essential to reverse the incapacity of NK cells (Figure 2)**.**

#### 5.3.1. Releasing Dominant Inhibition

The classical NK cell inhibitory receptors, KIRs, CD94/NKG2A, and LIRs, exert “dominant inhibition” to induce abortion of activation signals rather than trigger apoptosis [97,98]. Blocking these receptors showed promising anti-cancer ability in a mouse model; however, clinical trials did not generate the expected results. IPH2101 is an IgG4 monoclonal antibody that targets KIR2DL1/2/3. IPH2101, as a monotherapy, was evaluated in various clinical trials and was well tolerated; however, it failed to increase the anti-cancer efficacy in refractory multiple myeloma patients [99]. Infusion of IPH2101 reduced the expression of KIR2D inhibitory receptor on NK cells, which disrupted the “education” process of NK cells, leading to the decreased quantity and quality of fully functional NK cells [100]. Despite the discouraging results, clinical trials of KIRs antibodies in combination with other agents are still underway. For example, in a phase I trial, the combination of IPH2101 and lenalidomide as “dual immunotherapy” for treating relapsed/refractory multiple myeloma patients was well tolerated. Objective responses were observed in 5 of 15 patients, and the median progression-free survival was 24 months compared with 4.9 months in patients with lenalidomide alone. This trial suggested that IPH2101, as a promising combinational agent, warrants further investigation [101]. Another phase I study showed that the combination of the KIR2DL1/2/3 antibody lirilumab and PD-1 antibody nivolumab was tolerable in patients with advanced lymphoma and multiple myeloma, but the study was not conclusive on efficacy. Considering the limited number of patients in the trials, the researchers did not conclude the complete failure of this combination treatment, and they thought future strategies should focus on selecting better and more effective checkpoints [102].

HLA-E and HLA-G are ligands for NKG2A and LIR, respectively, and cancer cells upregulate the expression of these MHC-I molecules to avoid killing by NK cells [13]. NKG2A is expressed in over 50% of peripheral blood NK cells. The use of the anti-NKG2A antibody, monalizumab, could unleash NK cells and enhance anti-cancer immunity in combination with PD-1/PD-L1 axis blockade in a lymphoma mouse model [103]. Moreover, the researcher evaluated the combination of the NKG2A antibody monalizumab and anti-EGFR antibody cetuximab in treating patients with squamous cell carcinoma of the head and neck, a type of cancer with strong expression of HLA-E. Combination therapy with such anti-cancer agents amplifies the activation of NK cells by relieving the inhibition of CD94/NKG2A and enhancing the ADCC effect [103]. Therefore, the selection of an optimized combined therapy is required for specific patients.

#### 5.3.2. Blocking Immunotolerance Signal

TIGIT, LAG-3, and TIM-3 are co-inhibitory receptors expressed on NK cells and are regarded as feasible next-generation immune checkpoint therapies [104]. Ligands of these receptors are highly expressed on cancer cells and are associated with NK cell exhaustion and unfavorable prognosis. TIGIT competes with the activating receptor DNAM-1 for the ligands CD155 and CD112; thus, blocking TIGIT prevents the exhaustion of cancer-infiltrating NK cells and stimulates potent anti-cancer efficacy. The researchers also found that TIGIT-positive NK cells are mostly PD-1 negative, while T cells had double-positive expression, which indicated that TIGIT depressed the anti-cancer efficacy more specifically dependent on NK cells [105]. Although cancer-infiltrating NK cells have low expression of PD-1 (less than 10%), ongoing clinical trials are mostly designed in combination with PD-L1 antibody considering the ADCC effect [106]. In the phase II CITYSCAPE trial, the TIGIT antibody tiragolumab combined with PD-L1 antibody atezolizumab for the treatment of PD-L1high NSCLC patients showed a significant benefit with an objective response rate of 66% compared to 24% with atezolizumab alone [107]. These exciting results led to the US Food and Drug Administration approval of tiragolumab/atezolizumab as the first-line therapy for metastatic PD-L1-positive NSCLC patients without EGFR or ALK mutations. Other clinical trials using the tiragolumab/atezolizumab combination for treating solid tumors have also demonstrated promising effects [108]. Furthermore, blocking PD-1/PD-L1 signaling could markedly enhance the anti-cancer ability of NK cells and suppress the progression of cancer, suggesting targeting PD-1/PD-L1 axis on NK cells is a reasonable rationale for NK cell-based therapy [109].

#### 5.3.3. Targeting Emerging Inhibitory Checkpoints

There are some emerging targets for NK cell immune checkpoint therapy. Cytokine-induced STAT inhibitor (CIS), encoded by the Cish gene, is a negative regulator of IL-15 signaling through the inhibition of the JAK/STAT5 pathway [110]. Deleting the expression of the Cish gene could enhance the proliferation, IFN-γ production, and cytotoxicity of NK cells and inhibit solid tumor growth in mouse models. Cish gene deficiency combined with other checkpoint inhibitors or cytokines improves cancer metastasis control, indicating that CIS could act as a promising target to complement NK cell-based therapies [111].

Another newly discovered target is the signal regulatory protein α (SIRPα)-CD47 immune checkpoint. Through the stimulation of IL-2, NK cells upregulate the expression of SIRPα, which interacts with the inhibitory ligand CD47 expressed on cancer cells, delivering an inhibitory signal to NK cells. Under the precondition of eliminating the ADCC effect, IL-2-stimulated primary NK cells in conjunction with anti-CD47 antibody could rapidly eliminate K562 cells, providing a new strategy for immune regulation [112]. The PD-L1 and CD47 antibody simultaneous blocking “don’t find me” and “don’t eat me” signal mediated more effective anti-cancer immunity in a mouse model compared with single antibody treatment [113].

In addition, cancer-sensitized NK cells with a regulatory phenotype upregulate CD73 expression, which is correlated with a heavier cancer burden. CD73-positive NK cells undergo transcriptional reprogramming, highly expressing other inhibitory checkpoints, such as PD-1 and LAG-3, and suppressing the activity of CD4 T cells by secreting TGFβ and IL-10 via the STAT3 pathway [114]. Therefore, future studies should focus on the inducible checkpoint CD73 as a potential solution for activating NK cells and enhancing anti-cancer immunity in various solid tumors.

## 6. Conclusions

NK cells, as important immunoregulatory cells, play essential roles in cancer immunosurveillance. However, the functions and characteristics of NK cells are impaired or transformed during cancer progression. NK cells are functionally suppressed in the TME due to multiple immunosuppressive factors, especially TGFβ. Therefore, an increasing number of studies have been conducted to enhance the anti-cancer function of NK cells via cytokines and blocking antibodies. Technical limitations impede the development of NK cell therapy. However, technological advancement has facilitated NK cell generation, expansion, and genetic modification ex vivo, thus enhancing the anti-cancer properties of NK cell therapy. The therapeutic effects of NK cell therapy alone or in combination with other agents have been widely demonstrated in multiple clinical trials, and further preclinical studies are underway. Therefore, it is reasonable to believe that NK cell therapy could be a promising treatment option for cancer.

## Figures and Tables

**Table 1:** Human NK cells receptors and their ligands.

Receptor	Ligand
Activating Receptors	
NKG2D	MICA/B, ULBPs
DNAM-1(CD226)	CD112(nectin 2), CD155(PVR)
CD16	IgG Fc region
NKp46	HS GAGs, CFP
NKp30	HS GAGs, B7-H6, Galectin-3
NKp44	NKP44L, HS GAGs, MLL5, PCNA, PDGF-DD, Nidogen-1
Inhibitory Receptors	
KIRs	HLA-A, B, C(i.e., C1, C2, Bw4)
CD94/NKG2A	HLA-E
LIR-1	HLA-G
CD96, TIGIT	CD112, CD155
LAG3	MHC II
PD-1	PD-L1

MICA/B-MHC class I chain-related protein A/B; ULBP-Cytomegalovirus UL16-binding protein; PVR-Poliovirus receptor; HS GAGs-Heparan sulfate glycosaminoglycans; CFP-Complement factor properdin; MLL5-Mixed-lineage leukemia protein-5; PCNA-Proliferating cell nuclear antigen; PDGF-DD-Platelet-derived growth factor-DD; KIR-Killer immunoglobulin-like receptor; LIR-Leukocyte immunoglobulin-like receptor; TIGIT-T-cell immunoreceptor with Ig and ITIM domains; LAG3- Lymphocyte activation gene 3; PD-1-Programmed cell death protein-1.

## Data Availability

No new data were created or analyzed in this study. Data sharing is not applicable to this article.
